# Genome-wide analysis of the AP2/ERF gene family in *Tritipyrum* and the response of *TtERF_B2-50* in salt-tolerance

**DOI:** 10.1186/s12864-023-09585-x

**Published:** 2023-09-13

**Authors:** Xiaojuan Liu, Guangyi Zhou, Songshu Chen, Zhenzhen Jia, Suqin Zhang, Mingjian Ren, Fang He

**Affiliations:** 1 Guizhou Subcenter of National Wheat Improvement Center, Agronomy College, Guizhou University, Guiyang, 550025 China; 2 School of Life Sciences, Guizhou Normal University, Guiyang, 550025 China

**Keywords:** *Tritipyrum*, AP2/ERF, Salt-tolerance, *TtERF_B2-50*, Genome-wide, Expression patterns

## Abstract

The AP2/ERF transcription factor is widely distributed across the plant kingdom and plays a crucial role in various abiotic stress responses in plants. *Tritipyrum*, an octoploid resulting from an intergeneric cross between *Triticum aestivum* (AABBDD) and *Thinopyrum elongatum* (EE), is a valuable source of germplasm for incorporating superior traits of *Th. elongatum* into *T. aestivum*. With the recent availability of whole -genome sequences for *T. aestivum* and *Th. elongatum*, we explored the organization and expression profiling of *Tritipyrum* AP2/ERF genes across the entire genome. Our investigation identified 543 *Tritipyrum* AP2/ERF genes, which evolutionary analysis categorized into four major groups (AP2, DREB, ERF, and RAV), whose members share a conserved motif composition. These 543 *TtAP2/ERF* genes were distributed throughout 28 chromosomes, with 132 duplications. Synteny analysis suggests that the AP2/ERF gene family may have a common ancestor. Transcriptome data and Real-Time PCR expression profiles revealed 43 *TtAP2/ERF* genes with high expression levels in response to various salt stressors and recovery regimens. Tel2E01T236300 (*TtERF_B2-50*) was particularly salt stress-sensitive and evolutionarily related to the salt-tolerant gene *AtERF7* in *A. thaliana*. Pearson correlation analysis identified 689 genes positively correlated (R > 0.9) with *TtERF_B2-50* expression, enriched in metabolic activities, cellular processes, stimulus response, and biological regulation. Real-time PCR showed that *TtERF_B2-50* was highly expressed in roots, stems, and leaves under salt stress. These findings suggest that *TtERF_B2-50* may be associated with salt stress tolerance and may serve as a valuable foreign gene for enhancing salt tolerance in wheat.

**Supplementary Information:**

The online version contains supplementary material available at 10.1186/s12864-023-09585-x.

## Introduction

Soil salinization is a significant abiotic stress affecting approximately 8.31 × 10^8^ hm^2^ of soil resources globally, with an annual economic loss of $27.3 billion worldwide resulting from salt-induced agricultural yield reduction [1, 2]. While high-yielding fields have seen slow growth in recent years, there remains great potential for improvement in the vast number of low- and medium-yielding fields. Consequently, effectively utilizing saline and other underutilized fields to increase total food production is a crucial problem requiring resolution. Salt stress can lead to primary stresses, including osmotic stress and ion toxicity, and induce a series of secondary stresses such as oxidative stress and nutrient stress [3]. The accumulation of multiple stresses can impact cell growth and metabolic processes, ultimately affecting seed germination, seedling growth, and crop yield [4, 5]. To cope with salt stress, plants have evolved intricate mechanisms at the morphological-structural, physiological-metabolic, and molecular levels, including reducing leaf number and area, closing stomata, accumulating osmoregulatory substances, and compartmentalizing Na^+^ and Cl^−^, as well as scavenging reactive oxygen species and altering stress-responsive gene expression [6, 7]. The expression of stress-responsive genes is essential for the degree of improvement in morphological structure and physiological metabolic levels, and transcription factors play a vital regulatory role in this gene expression [8].

AP2/ERF (APETALA2/ethylene responsive factor) is a plant-specific transcription factor family that has been isolated in various plants such as *Arabidopsis thaliana*, *Oryza sativa*, *Sorghum bicolor*, *Brassica pekinensis*, *Phyllostachys edulis*, *Zea mays*, *Hordeum vulgare*, and *T. aestivum* [9, 10]. These transcription factors typically consist of a DNA-binding domain, a transcription regulation domain, an oligomerization site, a nuclear localization signal (NLS), and a DNA-binding domain. To classify the AP2/ERF-like transcription factors based on the number and binding sequences of their structural domains, Sakuma et al. (2002) identified five subfamilies, including AP2/ERF (APETALA2), ERF (Ethylene Responsive), and AP2/ERF (Ethylene Responsive). The AP2/ERF-like transcription factor family was classified into five subfamilies based on the number of conserved structural domains and binding sequences of the ERF-like transcription factors. The subfamilies include AP2/ERF (APETALA2), ERF (Ethylene responsive element binding factor), DREB/CBF (Dehydration responsive element/C-repeat), RAV (related to AB13/VP), and Soloist [11]. The ERF and DREB subfamilies are further subdivided into six subgroups (A1-A6, B1-B6), distinguished by the differences in conserved residues in their structural domains. While AP2/ERF, DREB, and ERF subfamilies have been extensively studied, little research has been conducted on the Soloist subfamily, despite the high conservation of its nucleotide sequences in most plants [12].

The AP2/ERF family is known for its involvement in abiotic stress responses, specifically drought, high salt, and low temperature stresses. These stresses activate signaling pathways such as phytohormones ABA, jasmonate (JA), ethylene (ET), and salicylic acid (SA), which in turn induce the expression of AP2/ERF transcription factor family genes. These factors then regulate downstream functional genes. Salt-responsive AP2/ERF transcription factors bind to promoter GCC-box and dehydration-responsive elements/C-repeat (DRE/CRT) elements, which are involved in MAPK and ethylene signaling pathways, to regulate gene expression [13]. Some AP2/ERF transcription factors negatively regulate salt response through the presence of transcriptional repressor domains like EAR. Rice *OsMAPKKK6* activates *OsMAPKK4* through phosphorylation, and *OsMAPKK4*, in turn, activates *OsMAPKK4* via phosphorylation [14, 15]. The transcription factor *OsSERF1* is phosphorylated by *OsMAPK5* under salt stress and is responsible for activating downstream transcription factor genes, namely *OsDREB2A* and *OsZFP179*. Overexpression of *OsDREB2A* promotes rice germination and enhances survival under salt stress, while overexpression of *OsZFP179* activates the expression of the *OsLEA3* gene in rice [16–18]. In addition, *OsSERF1* can bind to its own promoter and the upstream promoters of *OsMAPK5* and *OsMAPKKK6*, thereby enhancing their expression. Meanwhile, the Arabidopsis ERF transcription factor, *AtESE1*, plays a crucial role in mediating the ethylene pathway in response to salt stress. The key ethylene pathway transcription factor, *AtEIN3*, binds to the *AtESE1* promoter, which subsequently binds to the promoters of *AtRD29A*, *AtCOR15A*, and *AtP5CS2* to improve salt tolerance during seed germination and at the seedling stage [19]. The regulatory feedback mechanisms of AP2/ERF transcription factors merit further attention for understanding their regulatory networks.

*T. aestivum* is a moderately salt-tolerant crop, exhibiting higher salt tolerance than rice but lower salt tolerance than barley. In comparison, *Th. elongatum*, a closely related species to *T. aestivum*, can tolerate salt concentrations equivalent to those of seawater. The octoploid *Tritipyrum*, which results from intergeneric crosses between *T. aestivum* (AABBDD) and *Th. elongatum* (EE), offers a promising source of germplasm for introducing salt tolerance genes from *Th. elongatum* into *T. aestivum*. The complete sequencing of the genomes of *T. aestivum* and *Th. elongatum* lays a solid foundation for investigating the structural and functional features of srelevant genes [20, 21]. The aim of this study was to investigate the genomic structural features, chromosomal locations, gene duplication patterns, and evolutionary divergence of the AP2/ERF gene family in *Tritipyrum*, as well as to analyze the expression profiles of 43 *TtAP2/ERF* genes under salt stress conditions. Furthermore, we evaluated the expression levels and Pearson correlation of *TtERF_B2-50* during salt stress and recovery. The findings of this study can provide insights into potential strategies for enhancing plant salt tolerance.

## Material and methods

### Plant material

The artificially synthesized octoploid *Tritipyrum* consists of the A, B, and D genomes of *T. aestivum* and the E genomes of *Th. elongatum*. A stable offspring of a wide cross between *T. aestivum* and *Th. elongatum*, named 'Y1805', is salt-tolerant and was provided by Prof. Qingqin Zhang (formerly of Agronomy College at Guizhou University, Guiyang, China). 'Y1805' has been maintained in our laboratory through self-crosses. The *Tritipyrum* protein and nucleic acid sequences used for identifying AP2/ERF genes were obtained from the genome databases of *T. aestivum* and *Th. elongatum*. Phytozome 13, the Plant Genomics Portal, includes genome sequences of several plants, including *A. thaliana*, *H. vulgare*, *O. sativa*, *Z. mays*, and *Thinopyrum intermedium*. Meanwhile, the genome sequences of *Secale cereale* were obtained from the China National GeneBank Database (https://ngdc.cncb.ac.cn/gwh/Assembly/12832/show). To investigate transcriptome datasets available to the public, this study utilized information available at [https://www.ncbi.nlm.nih.gov/bioproject/PRJNA769794], with an accession number of PRJNA769794.

### Genomic in situ hybridization

The seeds were germinated on moist filter paper in Petri dishes at 25°C and then kept at 4°C for about 24 h before being transferred back to 25°C. Prior to fixation in Carnoy's solution, roots measuring 1–2 cm in length were excised and soaked in ice water for roughly 24 h. The mitotic chromosomes present in the root tips were observed under a microscope after fixation and staining with carbol fuchsin. For probes, the DNA of *Th. elongatum* was fluorescently labeled with fluorescein-12-dUTP using the nick translation method, while sheared genomic DNA from Chinese Spring (2n = 42) was utilized as blocking DNA. The slides were counterstained using propidium iodide (0.25 mg/mL) in Vectashield (Vector Laboratories, USA) mounting medium.

### Identification of the AP2/ERF genes in *Tritipyrum*

The consensus protein sequences of the AP2/ERF domain hidden Markov model (HMM) (PF00847) were retrieved from the Pfam database (https://www.ebi.ac.uk/interpro/), while a library of search files was compiled from 31 reported *A. thaliana* AP2/ERF gene family sequences obtained from the UniProt database (www.uniprot.org). Using the AP2/ERF domain sequences of *A. thaliana* as query sequences, we utilized the Basic Local Alignment Search Tool algorithm (BLASTP) to identify *Tritipyrum* AP2/ERF (*TtAP2/ERF*) proteins. After removing duplicates, the remaining candidate sequences were validated using the Pfam database and the SMART tool (http://smart.embl-heidelberg.de/) [22]. Physical and chemical characteristics of the genes were generated using ExPASy (http://web.expasy.org/protparam/).

### Phylogenetic analyses and conserved motif determination

To construct phylogenetic trees for *A. thaliana* AP2/ERF proteins, several amino acid sequences were aligned using the ClustalX 2.0 program. The UniProt database provided the necessary protein sequences. For *Tritipyrum* and *A. thaliana*, the NJ method with the Poisson model and 1000 bootstrap replications were used as specific parameters to construct the phylogenetic trees. Using the online tool MEME, conserved motifs in *TtAP2/ERF* proteins were identified with optimum mode width set to 6 to 200 and a maximum of 10 motifs. Phylogenetic trees were displayed, edited, and colored using FigTree and iTOL tools, which are available at http://iTOL.embl.de/.

### Chromosomal distribution and gene duplication of the *TtAP2/ERF* genes

The *TtAP2/ERF* genes were mapped to *Tritipyrum* chromosomes using the method described by Liu et al. To analyze the gene duplication events, we employed the multiple collinear scanning toolkit (MCScanX) with default parameters. To perform the all-vs-all protein sequence comparisons required for MCScanX, we used DIAMOND v0.8.25 with the parameters –max-target-seqs 5 and –evalue 0.00001 [23].

### Plant growth conditions and stress treatments

The Y1805 seeds were germinated in a growth chamber under controlled conditions with a relative humidity of 75% and a 20/15°C light–dark photocycle. The seedlings were grown in 1/2 Hoagland's solution on a floating board with a 16/18-h light/dark cycle, an irradiance of 400 μmol m^−2^s^−1^, and the same temperature and relative humidity as the germination chamber. The culture solution was replaced three times a week. At the two-leaf stage (14 days after germination), salt stress treatments (1/2 Hoagland's solution plus 250 mM NaCl) were initiated, and *Tritipyrum* root, stem, and leaf samples of uniform size were collected after 5 h of exposure to salt stress (T1). The plant materials were then recovered in 1/2 Hoagland's solution without NaCl after 24 h of salt stress, and the second sample was collected one hour following recovery (T2). CK1 and CK2 were grown in normal conditions (1/2 Hoagland's solution without NaCl) as parallel controls. For Real-Time PCR and gene cloning, all tissue samples were immediately frozen in liquid nitrogen and stored at -80 °C. Each tissue sample consisted of at least ten seedlings from each of three biological replications, which were combined.

### Expression analysis and Real-Time PCR validation of AP2/ERF genes under salt stresses and recovery

This study utilized transcriptome datasets at [https://www.ncbi.nlm.nih.gov/bioproject/PRJNA769794, accession number: PRJNA769794]. The RNA-Seq data sets were employed to examine the expression of *Tritipyrum* AP2/ERF genes during salt stress and recovery. The sequencing data utilized the fastp software (https://github.com/OpenGene/fastp) to eliminate reads containing adaptor contamination, low-quality bases, and undetermined bases by using the default parameters. The resulting high-quality reads were aligned to the combined genomes of *T. aestivum* (IWGSCv2.0) and *Th. elongatum* (v1.0). To create the reference genome index, Bowtie (v2.2.3) was employed, and HISAT (v2.2.0) was utilized to align the filtered reads to the composite reference genome. The number of reads corresponding to AP2/ERF genes was quantified using FeatureCounts version 1.5.1, and the read abundance was computed as transcripts per kilobase (TPM) [24]. The results were transformed and visualized using the R Circlize package (https://CRAN.R-project.org/package=circlize). Gene ontology (GO) annotation was achieved by mapping GO term using the BLAST2GO tool (Biobam Bioinformatics S.L., Valencia, Spain, http://blast2go.com/b2ghome/about) with an e-value threshold of 10^–6^. To design primers, Primer 5.0 was used (Supplementary Table S2). The *Actin* gene (accession number: AB181991) was utilized as an internal control, which was uniformly expressed at each growth stage and nearly in all tissues. The detection of three technical repeats of the three biological repeats was calibrated using the *Actin* gene (accession number: AB181991), and the 2^−ΔΔCt^ method was employed to determine the expression.

### Statistical analysis

The study data were analyzed using SPSS software (IBM Corporation) through ANOVA. Mean values were compared using the least significant difference (LSD) test at a significance level of 0.05. Histograms were generated with Origin 8.0 (OriginLab Corporation, Northampton, Massachusetts, USA).

## Results

### Identification of the *TtAP2/ERF* genes in *Tritipyrum*

**Fig. 1:**
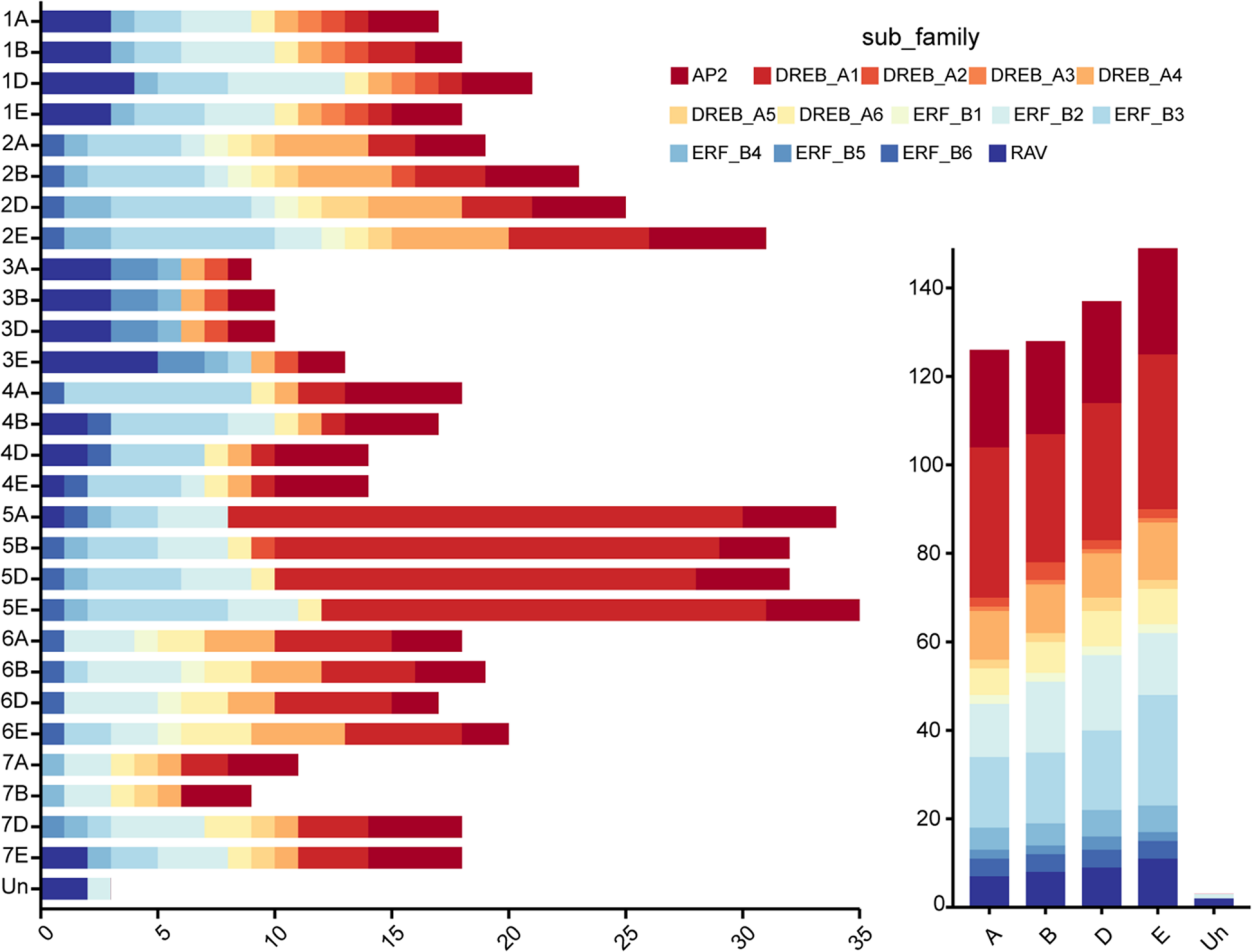
Chromosomal and subgenomes distribution of AP2/ERF genes in *Tritipyrum*

The cytogenetic analysis of *Tritipyrum* 'Y1805' indicated that it has 56 chromosomes, consisting of 42 from *T. aestivum* (blue) and 14 from *Th. elongatum* (green), as illustrated in Figure S1. To identify AP2/ERF genes in the *Tritipyrum* genome, we employed the hidden Markov model (HMM) and BLASTp approaches, which yielded a total of 543 non-duplicated sequences. We subsequently renamed these genes based on their chromosomal location in *Tritipyrum* (Supplementary Table S1). Our findings revealed that the majority of these AP2/ERF genes were located in the E subgenomes and fifth homologous groups, as shown in Fig. 1. The predicted AP2/ERF proteins in *Tritipyrum* exhibited substantial variation in both length and molecular weight (MV). Specifically, the length of the encoded proteins ranged from 90 (Tel2E01T293000.1) to 1422 (TraesCS2D02G511600.2) amino acids (aa), while the MV ranged from 9.87 (Tel2E01T293000.1) to 157.95 (TraesCS2D02G511600.2). Additionally, the isoelectric points (PIs) of these proteins varied between 4.1 (Tel1E01T375200.1) and 12.4 (TraesCS5B02G311300.1), as provided in Supplementary Table S1.

### phylogenetic analysis, and motif composition of the *TtAP2/ERF* gene

**Fig. 2:**
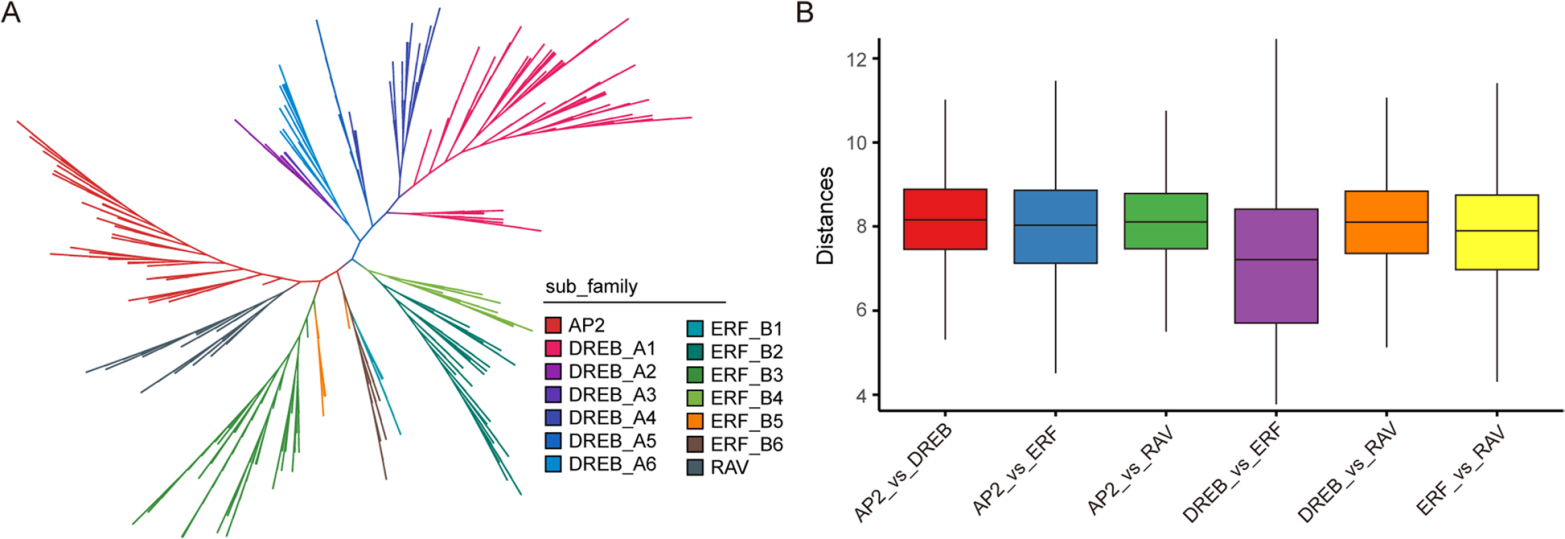
phylogenetic relationships and distance among the AP2/ERF proteins from *Tritipyrum* and *A. thaliana,* **A** Phylogenetic relationships among 574 AP2/ERF proteins from *Tritipyrum* and *A. thaliana*; **B** genetic distance among the different clades of AP2/ERF genes. The box plot shows the median (black line), interquartile range (box), and maximum and minimum scores (whiskers) of each data set

To construct a phylogenetic tree and classify the AP2/ERF family in *Tritipyrum*, we used 574 potential AP2/ERF structural domains identified in *A. thaliana* and *Tritipyrum* (Fig. 2A, Fig. s2). *Tritipyrum*'s AP2/ERF family was classified into four major groups (AP2, DREB, ERF, and RAV) based on their AP2/ERF classification and primary amino acid sequence characteristics in *A. thaliana*, with no subgroup Soloist identified. The DREB and ERF groups are the two largest subgroups of *Tritipyrum*'s AP2/ERF transcription factor family, which were further subdivided into six subfamilies each. The WLG motif was present in all *Tritipyrum* and *A. thaliana* DREB subfamily factors, and most subfamily factors had the YRG element (Fig. s2). The *Tritipyrum* and *A. thaliana* DREB subfamily factors were highly conserved, especially the DREB_A3 subgroup (Fig. s3). While ERF_B1 exhibited greater variation than ERF_B2, ERF_B3, ERF_B4, and ERF_B5 (Fig. s3), *Tritipyrum* and *A. thaliana* exhibited the smallest genetic separation between DREB and ERF (Fig. 2A, B).

### Chromosomal distribution, gene duplication and synteny analysis of the *TtAP2/ERF* gene

**Fig. 3:**
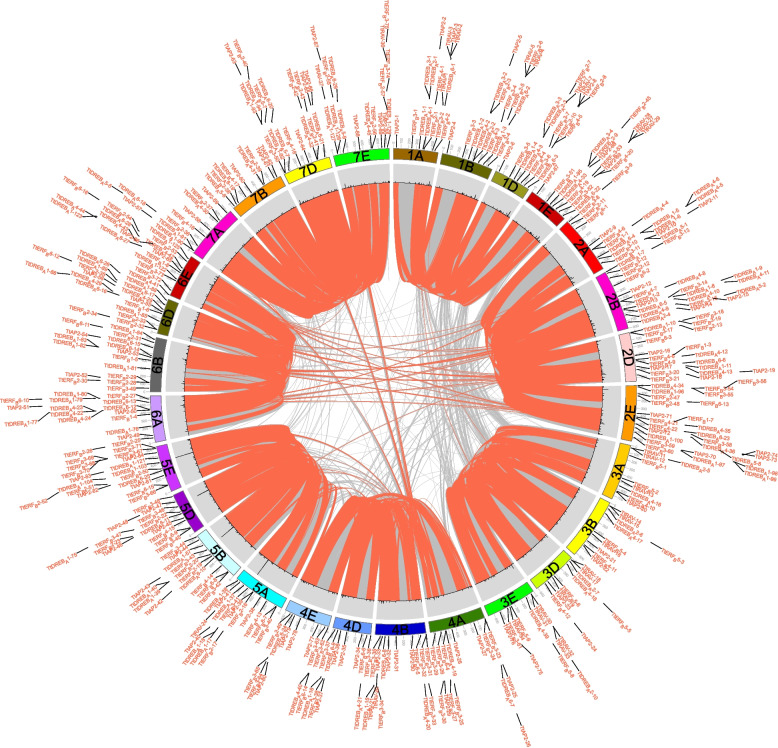
distribution, duplication and synteny analysis of AP2/ERF genes in *Tritipyrum,* Collinear correlations of AP2/ERF in *Tritipyrum* genomes are displayed by Circos. *Tritipyrum* chromosomes are colored according to the inferred ancestral chromosomes following an established convention. In the center, the relative map position of 540 AP2/ERF genes is shown on each of the 28 *Tritipyrum* chromosomes

The genome chromosomal placements of the *TtAP2/ERF* genes were investigated, revealing that out of the 543 genes, 540 were found localized to 28 chromosomes, with *TtRAV-25*, *TtRAV-26*, and *TtERF_B2-46*absent from the chromosome localization map due to their anchoring on scaffolds (Fig. 3; Supplementary Table S1). The majority of the *TtAP2/ERF* genes were dispersed in the second (18.05%) and fifth (24.5%) homologous clusters, whereas relatively few were present in the third and seventh homologous groups (Figs. 1 and 3; Supplementary Table S1). The majority of *TtAP2/ERF* genes were located towards the chromosome's ends, while only a few were close to the center (Fig. 3). These findings indicate that the distribution of AP2/ERF genes on chromosomes was nonrandom and unequal. Furthermore, the gene duplication analysis indicated that the *TtAP2/ERF* genes underwent both tandem and segmental duplications. Specifically, 32 tandem duplications and 72 segmental duplication gene pairs were discovered (Fig. 3), suggesting that some genes have several copies due to multiple replications within the *T. aestivum* genome. Most homologous genes are dispersed in the same homologous groups, with only a few presents in the fourth, fifth, and seventh homologous groups, consistent with the natural translocations that occurred throughout *T. aestivum*'s development and evolution.

### Evolutionary analysis of the AP2/ERF Families in several different species

**Fig. 4:**
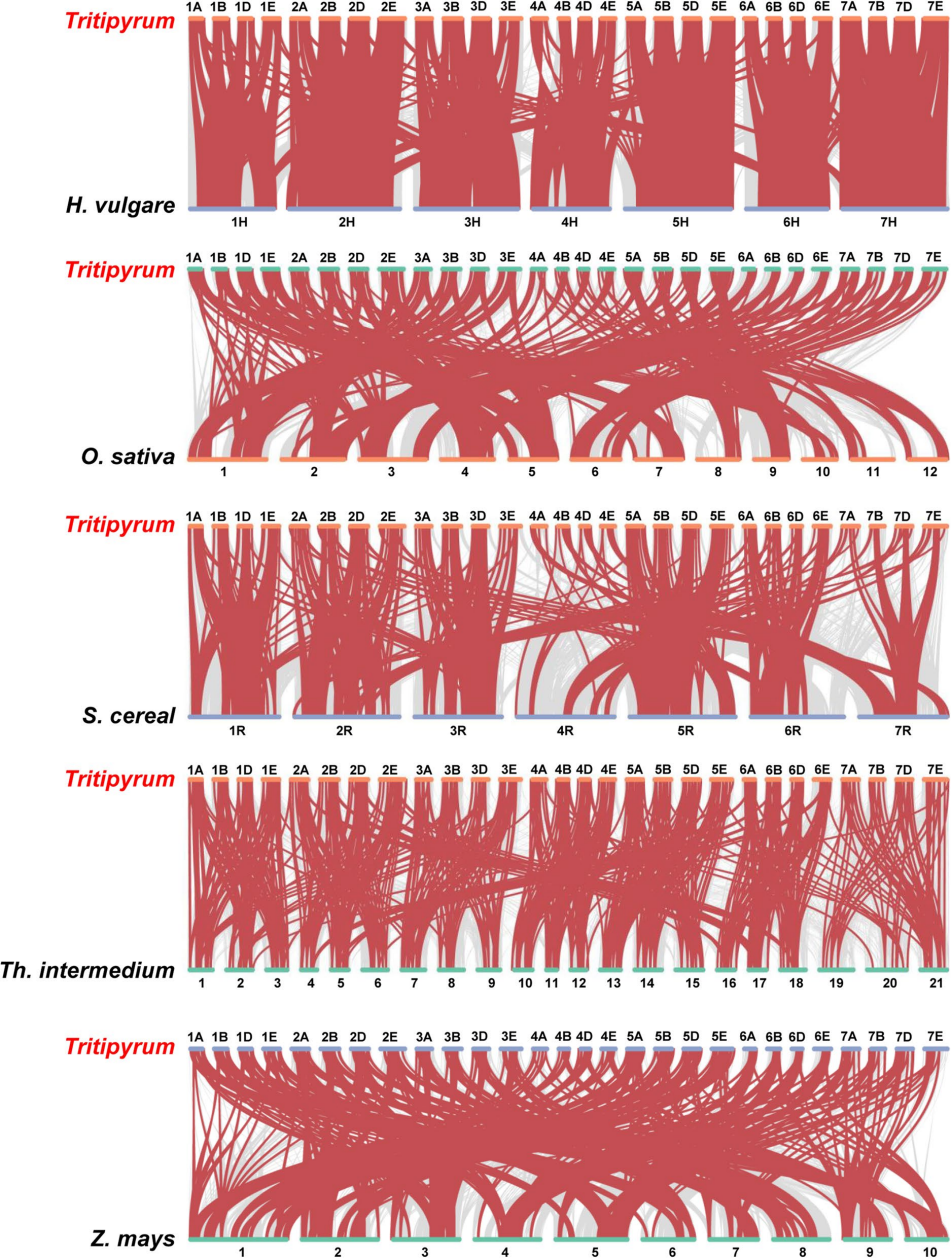
Synteny analyses between *Tritipyrum* and five representative plant species, Gray lines in the background indicate collinear blocks within *Tritipyrum* and other plant genomes, while red lines highlight syntenic AP2/ERF gene pairs

The AP2/ERF gene family is present in several plant species, including *Tritipyrum*, *H. vulgare*, *O. sativa*, *S. cereal*, *Th. intermedium*, and *Z. mays*. Through an examination of the syntenic relationships among these species, it is possible to gain insight into the evolutionary relationships between these genes. To date, five distinct classes of syntenic connections have been identified (Fig. 4). Among these species, *TtAP2/ERF* genes were found to have the highest number of syntenic linkages with *Th. intermedium* (344), followed by *Z. mays* (227), *S. cereal* (175), *O. sativa* (149), and *H. vulgare* (143) (Fig. 4). Collinear pairs between *Tritipyrum* and the other five species suggest that these orthologous pairs may have been present prior to their divergence. In addition, specific AP2/ERF collinear gene pairs found in *Tritipyrum* and *S. cereal* were linked to highly conserved syntenic blocks, including over 500 collinear sites. These findings suggest that *Tritipyrum* and the other five plant species may have an evolutionary connection that could explain the observed similarities in trend between *Tritipyrum* and *H. vulgare*. Notably, several *TtAP2/ERF* genes were related to at least three syntenic gene pairs, indicating their crucial role in the evolution of the AP2/ERF gene family. These results demonstrate the high conservation of the *TtAP2/ERF* gene family and support the idea that there may have been a common ancestor from which the *TtAP2/ERF* genes of multiple plants arose. Additionally, the *TtAP2/ERF* genes were found to be more closely related to those of *Z. mays* than those of *H. vulgare*.

### Expression of *TtAP2/ERF* genes under salt stresses and recovery

**Fig. 5:**
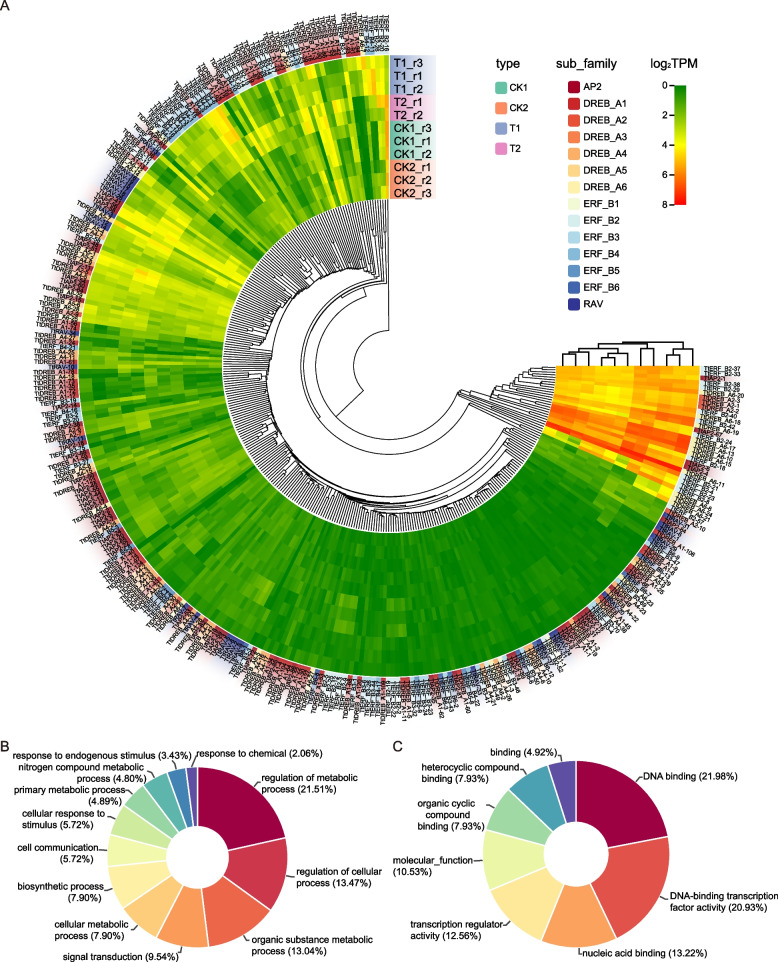
Expression patterns of *TtAP2/ERF* genes under salt stresses and recovery treatments, **A** Hierachical clustering of expression profiles of 310 *TtAP2/ERF* genes were expressed in 11 samples including salt stress and recovery treatment. **B** and **C** the BP (B) and MF (C) analysis of 310 expression genes

To investigate the effect of various salt stresses and recovery treatments on the expression of *TtAP2/ERF* genes, we analyzed the transcriptional levels of all 543 genes belonging to this family. Among the 11 samples tested, 310 *TtAP2/ERF* genes were expressed consistently, and 145 genes demonstrated constitutive expression (TPM > 1 in all samples). Cluster analysis revealed no significant correlation between subfamily types and response to salt stress and recovery treatments for the 543 *TtAP2/ERF* genes (Fig. 5A). We found that 233 *TtAP2/ERF* genes were not expressed in any of the detected samples, suggesting that they may be pseudogenes or not respond to salt stress and recovery. Gene Ontology (GO) annotation of the 310 expressed genes showed that their biological processes (BP) and molecular functions (MF) included regulation of metabolic and cellular processes, organic substance metabolic process, signal transduction, cellular metabolic process, and DNA binding. The genes had DNA-binding transcription factor activity, nucleic acid binding, transcription regulator activity, and molecular function (Fig. 5B and C).

**Fig. 6:**
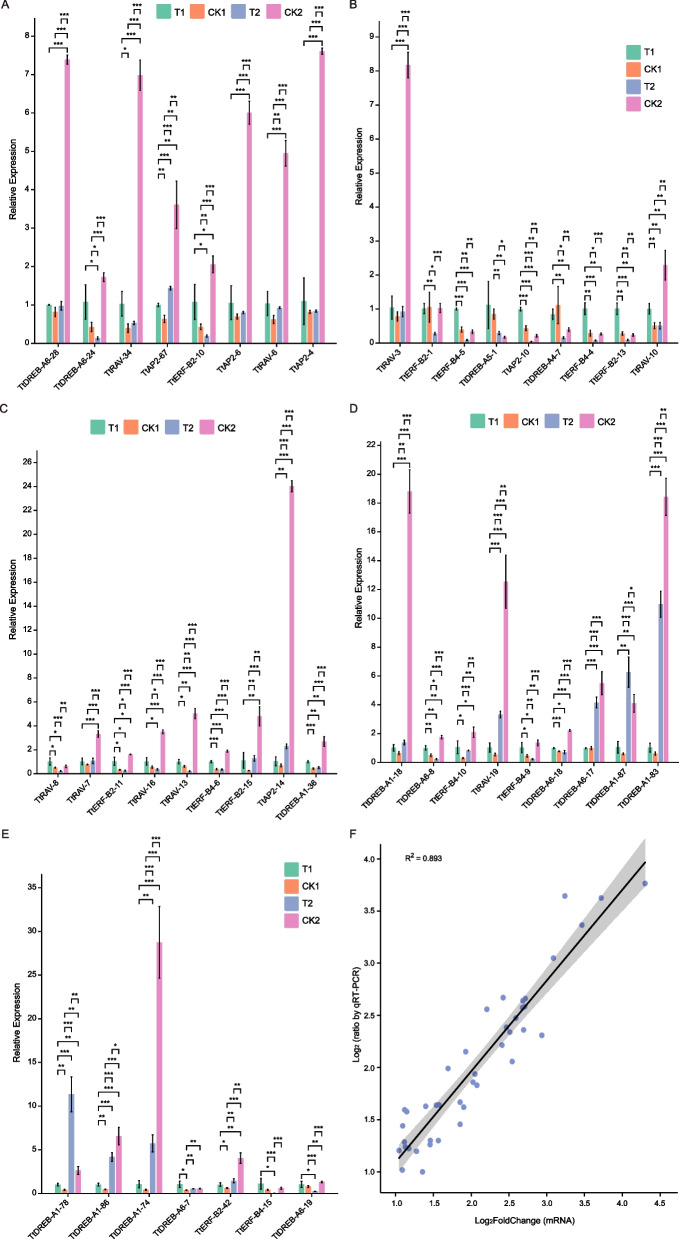
**A-E** Expression analysis of 43 AP2/ERF genes in eleven samples by Real-Time PCR. Data were normalized to β-actin gene and vertical bars indicate standard deviation. **F** The relationships between Real-Time PCR and transcriptional of 42 up-regulated expression genes. Values are the log_2_ratio (salt stress or recovery treatment /CK treatment) for genes. The determine coefficient (r^2^) is indicated in the figure. All Real-Time PCR reactions were performed in three biological replicates. Asterisk, double and triple asterisks indicate significant differences (*p* < 0.05, 0.01 and 0.001, respectively) between groups

To validate our transcriptome data, we selected 42 *TtAP2/ERF* members out of the 310 *Tritipyrum* AP2/ERF genes whose mRNA levels remained consistently high (log_2_FoldChange > 1) during various salt stressors and recovery regimens. We designed primers specific to these 43 genes (Supplementary Table S2) and conducted further quantitative Real-Time PCR tests to investigate their expression patterns in response to different salt stress and recovery treatments. We found that the Real-Time PCR results were comparable with the transcriptome data, as shown in Fig. 6A-E. The trends in gene expression were significantly similar (r^2^ = 0.893) to those from the transcriptome data, confirming the validity of our transcriptome results (Fig. 6F).

### Expression patterns and correlation analysis of *TtERF_B2-50*

**Fig. 7:**
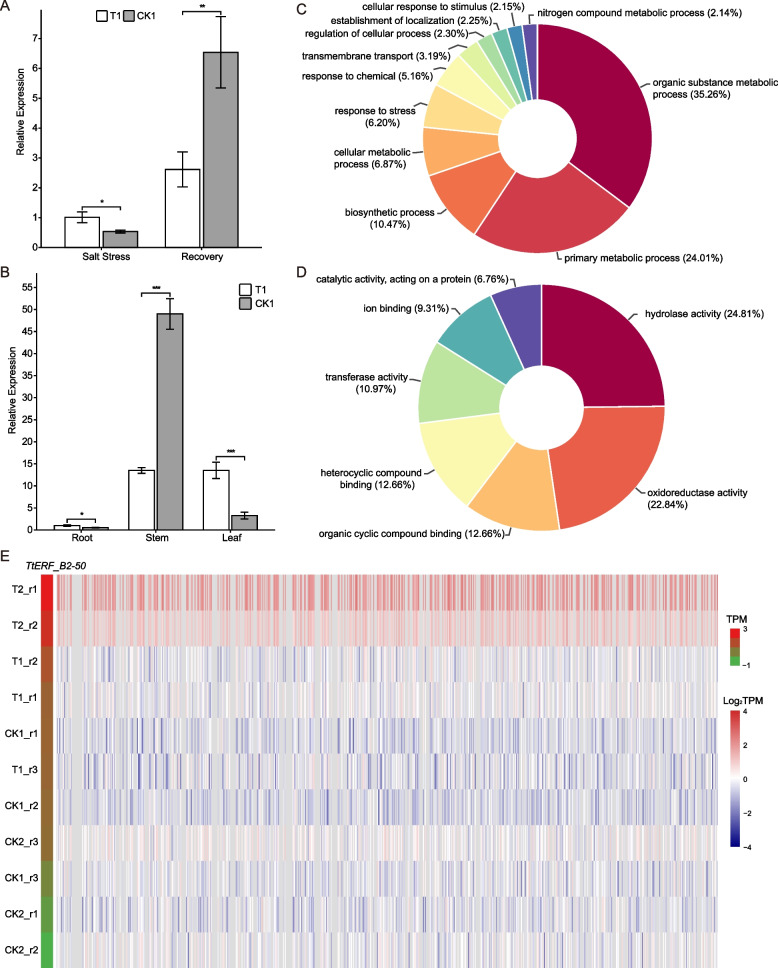
Expression patterns and correlation of *TtERF_B2-50*, **A** Relative expression levels of *TtERF_B2-50* in roots under salt stress and recovery conditions; **B** Relative expression levels of *TtERF_B2-50* in roots, stems, and leaves under salt stress; **C** and **D**: the BP (C) and MF (D) analysis of 689 positively related to *TtERF_B2-50* expression genes; E: six hundred eighty-nine genes positively related (R > 0.9) with *TtERF_B2-50* expression, each row represents a sample and each column represents a gene

Previous studies have demonstrated that the transfer of the *AtERF7* (AT2G47520) gene into mutant Arabidopsis plants resulted in enhanced salt tolerance and resistance to osmotic stress. Furthermore, these plants exhibited increase d superoxide content and greater capacity to withstand hypoxic stress. Interestingly, both the *TtERF_B2-50* (Tel2E01T236300) gene in the *Tritipyrum* AP2/ERF family and the *AtERF7* gene belong to the same evolutionary branch (Fig. S2) and are up-regulated in response to various salt stresses and recovery treatments (Fig. 5). To investigate the spatiotemporal expression pattern of *TtERF_B2-50*, we conducted Real-Time PCR analysis of its expression levels in roots under salt stress and recovery conditions, as well as in various tissues. Our results showed that the expression level of *TtERF_B2-50* in roots was significantly higher (1.89-fold) during salt stress than under control conditions (Fig. 7A), consistent with the transcriptome data. Moreover, when Y1805 was exposed to salt stress, the relative expression level of *TtERF_B2-50* was highest in the stems, followed by leaves and roots (Fig. 7B).

To investigate the biological processes associated with *TtERF_B2-50* expression during salt stress and recovery treatments, a Pearson correlation analysis was conducted on *TtERF_B2-50* and other genes from the transcriptome data. Results indicated a positive correlation between *TtERF_B2-50* expression and 689 genes (R > 0.9), with particularly high levels of expression observed in T1 and T2 samples (Fig. 7E). Gene ontology (GO) analyses revealed that the genes involved in key biological processes (BP) and molecular functions (MF) such as organic substance metabolic process, primary metabolic process, biosynthetic process, cellular metabolic process, response to stress, hydrolase activity, oxidoreductase activity, organic cyclic compound binding, heterocyclic compound binding, and transferase activity were positively correlated with *TtERF_B2-50* expression (Fig. 7C and D). Taken together, these results suggest that *TtERF_B2-50* is linked to abiotic stress tolerance in plants.

## Discussion

The AP2/ERF family of plant transcription factors, one of the largest families, has been extensively studied for its evolution, function, and response to abiotic stress across many plant species. In this study, we identified 543 members of the AP2/ERF gene family in *Tritipyrum*. The number of AP2/ERF transcription factors and their subfamilies varies widely between plant species, with the ERF and DREB classes comprising more than half of the AP2/ERF transcription factors. However, the Solosist-like subgroup is rare, with most plant species having only one member or none at all. Notably, rice lacks any members of this subfamily. Like rice, *Tritipyrum*'s 543 AP2/ERF genes are classified into four groups (AP2, DREB, ERF, and RAV), but they do not include any Solosist-like subgroups. The AP2/ERF gene family, one of the largest families of plant transcription factors, has been extensively studied in various plant species for its evolution, function, and response to abiotic stress. This study found that *Tritipyrum* contains 543 members of the AP2/ERF gene family. The number of AP2/ERF transcription factors and their subfamily members vary among plant species, with ERF and DREB classes representing more than fifty percent of the AP2/ERF transcription factors [25–27]. In contrast, the number of Solosist-like AP2/ERF transcription factors is meager, with most plant species possessing only one or none, as is the case with rice [28]. *Tritipyrum*'s AP2/ERF genes are classified into four groups (AP2, DREB, ERF, and RAV), similar to rice, but without the Solosist subgroup. Gene duplication is a common phenomenon that occurs through various mechanisms. The three most frequent types of gene duplication are whole genome duplication, tandem duplication, and segmental duplication [29]. In most plants, genome-wide or segmental duplication events have occurred, resulting in large-scale chromosome doubling and the retention of numerous chromosomes doubling segments in their genomes [30]. Gene duplication can lead to the expansion of gene families due to evolutionary processes such as polyploidy and duplication events [31, 32]. However, duplicated genes may have different expressions, and the function of new members may be modified by point mutations in coding DNA sequence regions or regulatory sites [33, 34]. The study investigated the synteny of AP2/ERF genomes in *Tritipyrum*, *H. vulgare*, *O. sativa*, *S. cereal*, *Th. intermedium*, and *Z. mays*. *Tritipyrum* and *Th. intermedium* exhibited the most syntenic linkages, indicating a close evolutionary relationship that is consistent with traditional Gramineae classifications. Conversely, the separate genera *Tritipyrum* and *S. cereal* had the fewest syntenic links. Among the five sets of syntenic relationships, 1–22 AP2/ERF genes were shared by at least three species, which could provide insight into the evolution of AP2/ERF genes across species.

Salinity is a significant environmental threat to crop production due to its adverse impact on plant performance, which interferes with cellular metabolism by increasing osmotic stress and the accumulation of harmful ions within plant cells [4]. Unlike the operation of single functional genes, transcription factors (TFs) can regulate multiple downstream salt stress response genes, and overexpression of salt tolerance TFs has been shown to enhance plant tolerance to salt. Plant genes encoding TFs are differentially expressed as part of their complex stress response mechanisms, with bHLH, bZIP, MYC, NAC, WRKY, and AP2/ERF among the TFs associated with salt tolerance pathways [35–38]. AP2/ERF genes have been discovered in Arabidopsis, rice, maize, and wheat in response to various environmental stresses [25–27, 33]. Tissue-specific expression of AP2/ERF genes may affect the growth and development of target tissues and organs by regulating transcriptional processes [39]. The DREB and ERF subfamilies of AP2/ERFs play crucial regulatory roles in plant responses to abiotic stress and are implicated in the regulation of a complex network of plant stress response signals. Studies have shown that some AP2/ERFs can be induced to express rapidly and continuously under stressful conditions, whereas other processes respond more slowly, suggesting that there may be two stress response modes that either depend on or do not depend on hormone signaling pathways, with some cross-talk between them [40]. Previous research has demonstrated that AP2/ERFs play a significant role in regulating plant hormone-mediated stress responses, such as abscisic acid (ABA), ethylene (ET), gibberellin (GA), cytokinin (CTK), and brassinolide (BR) [41–43]. Specifically, HRD (hardy), a member of the DREB-A4 family, and *ERF53*, *RAP2.4*, and *TG/RAP2.4a*, members of the DREB-A6 family, have been shown to play critical roles in drought and salt tolerance regulation [44–47]. In Arabidopsis and rice plants, HRD overexpression has been shown to enhance drought and salt tolerance [44]. TG (translu-cent green) overexpression resulted in leaf cell water content increases and vitrification of plant leaves, as well as regulation of ascorbate peroxidase (APX) gene expression to protect against photooxidative stress caused by reactive oxygen species (ROS) [47, 48]. Apart from Arabidopsis, *O. sativa*, *Z. mays*, *Lycopersicon esculentum*, and *Vitis vinifera*, DREB1 and DREB2 transcription factors have been extensively researched [49, 50]. In this study, 310 *TtAP2/ERF* genes were identified in the root tissues of *Tritipyrum* that significantly responded to salt stress induction, with 145 *TtAP2/ERF* genes exhibiting constitutive expression. It is possible that these *TtAP2/ERF*s regulate cellular basal life processes. Further examination of 43 *TtAP2/ERF* genes was conducted due to their potential significance in the salt stress response. The expression profiling and Real-Time PCR analysis revealed that these 43 *TtAP2/ERF*s may positively regulate the salt stress response in *Tritipyrum* root tissues. To better understand the precise biological activities of these *TtAP2/ERF*s, transgenic experiments will be conducted. Additionally, their potential application in genetic engineering for enhancing crop stress tolerance and other agronomic traits will be investigated.

Extensive research has been conducted on *A. thaliana*'s response to salt stress, yielding substantial insights into the mechanisms underlying plant stress responses. *AtERF71/HRE2*, a member of the Arabidopsis AP2/ERF family, has emerged as a crucial regulator of osmotic and hypoxic stress responses in plants. Research indicates that the level of *AtERF71/HRE2* mRNA transcripts increased significantly in response to anoxia, NaCl, mannitol, ABA, and MV stress, emphasizing its pivotal role in the plant's stress response. Loss-of-function mutants of *AtERF71/hre2* exhibited heightened sensitivity to osmotic stresses such as high salt and mannitol and increased ROS levels under high salt exposure. Conversely, *AtERF71/HRE2*-overexpressing transgenic plants showed superior stress tolerance to salt, mannitol, flooding, and MV, as well as lower ROS levels under high salt treatment [51]. These results suggest that *AtERF71/HRE2* plays a crucial role in plant stress response and may serve as a potential target for crop improvement in the face of abiotic stresses. With noteworthy, the *TtERF_B2-50* gene in the AP2/ERF gene family of *Tritipyrum* is evolutionarily related to the *AtERF71/HRE2* gene in *A. thaliana*, and is up-regulated in response to various salt stresses and recovery treatments. Remarkably, our study showed that the relative expression of *TtERF_B2-50* was highest in the stems of *Tritipyrum* under salt stress, followed by leaves and roots. We also observed that the expression of *TtERF_B2-50* was significantly higher in the entire plant in response to salt stress and recovery compared to the control. These results are consistent with our transcriptome data and previous research [25], supporting the proposition that *TtERF_B2-50* is expressed throughout the plant to aid in coping with salt stress. Additionally, our analysis indicated that the strong positive correlation genes of *TtERF_B2-50* are involved in essential biological processes such as metabolic and cellular processes, response to stimulus, biological regulation, and regulation of biological process. As a result, *TtERF_B2-50* exhibited a highly sensitive and robust expression throughout the entire plant, enhancing the plant's tolerance to salt stress.

Soil salinization poses a significant challenge, as it negatively impacts seed germination, seedling growth, and crop yield. However, *Th. elongatum*, a species closely related to *T. aestivum*, can thrive in salt concentrations akin to seawater. *Tritipyrum*, an intergeneric hybrid of *T. aestivum* and *Th. elongatum*, plays a vital role in introducing salt tolerance genes from *Th. elongatum* into *T. aestivum* [52, 53]. Stress-responsive genes are primarily involved in enhancing salt tolerance in plants, and their products help repair various salt-induced primary and secondary stress factors. In contrast to individual functional genes, transcription factors can modulate a series of downstream target genes, regulating physiological and biochemical processes in response to salt stress [54, 55]. In this study, we conducted a comprehensive evaluation of the AP2/ERF family in *Tritipyrum* using bioinformatics techniques, including phylogenetic, motif, and correlation analyses. As part of our transcriptomic inquiry into *Tritipyrum*'s response to salt stress, we conducted RNA-seq to identify AP2/ERF transcription factors involved in the plant's reaction. Using the Arabidopsis salt stress response-related gene *AtERF71/HRE2* as a reference gene, we identified the *TtERF_B2-50* gene and analyzed its expression levels under salt stress and recovery. It is worth noting that the plant's response to salt stress is a complex process involving multiple genes and pathways. The *TtAP2/ERF* transcription factors that we screened in this study have not been characterized transgenically, highlighting the need to investigate their interactions with other proteins and downstream target genes. Understanding the mechanisms underlying salt tolerance involving transcription factors and identifying candidate genes related to these factors is essential for genetic engineering efforts to develop salt-tolerant crops. By doing so, we can develop crops with enhanced tolerance to soil salinity, enabling more sustainable food production in areas affected by soil salinization.

## Conclusions

This study thoroughly investigated the AP2/ERF gene family in *Tritipyrum*. We analyzed the motif compositions of 543 full-length AP2/ERF genes in the same groups and subgroups, finding remarkable similarity. Our analysis of synteny and phylogenetic comparisons of AP2/ERF genes from several plant species sheds light on the evolutionary characteristics of these genes in *Tritipyrum*. We identified 43 *TtAP2/ERF* genes that play a crucial role in salt stress response in *Tritipyrum* based on their expression patterns in diverse tissues and in response to salt stress and recovery treatments. Moreover, *TtERF_B2-50* is a potential target gene for enhancing wheat's salt tolerance through biotechnology or molecular breeding. These findings provide valuable insights into the biological functions of specific AP2/ERF genes in *Tritipyrum*.

### Supplementary Information

**Additional file 1.** 

**Additional file 2.** 

**Additional file 3.**

**Additional file 4.** 

**Additional file 5.** 

## Data Availability

The datasets generated and/or analyzed during the current study are available in the National Center for Biotechnology Information repository, [https://www.ncbi.nlm.nih.gov/bioproject/PRJNA769794, accession number- PRJNA769794].
