# Supplementary figures and images for: Genome-wide analysis of the AP2/ERF gene family in Tritipyrum and the response of TtERF_B2-50 in salt-tolerance
Source: BMC Genomics. 2023 Sep 13;24:541. doi: 10.1186/s12864-023-09585-x (PMC10498623; doi:10.1186/s12864-023-09585-x)

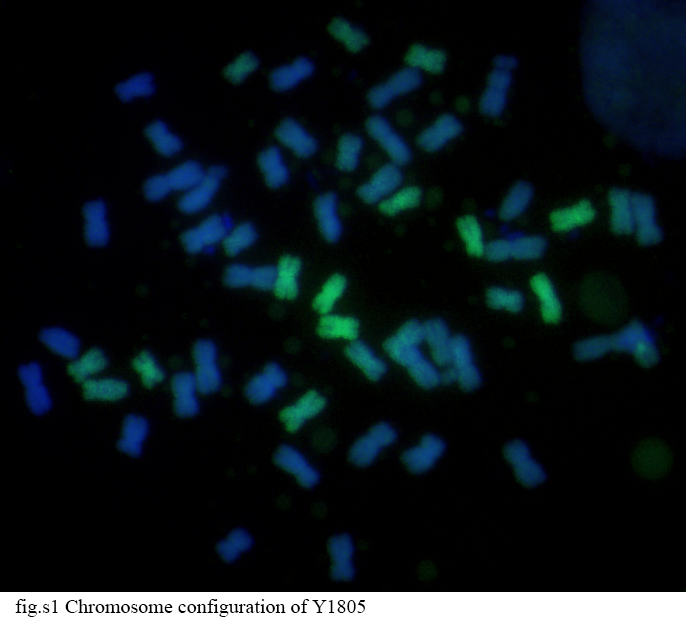

Supplement: Supplementary file 1 — Additional file 1. [file 12864_2023_9585_MOESM1_ESM.tif]

fig.s2 Phylogenetic trees of AP2\_ERF gene family in *Tritipyrum* and *A. thaliana*

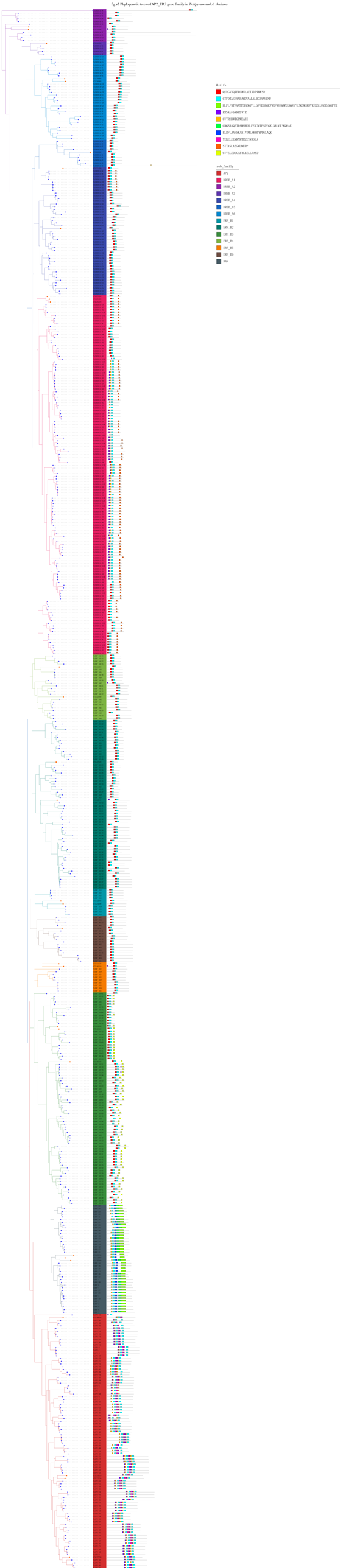

Supplement: Supplementary file 3 — Additional file 3. [file 12864_2023_9585_MOESM3_ESM.pdf]

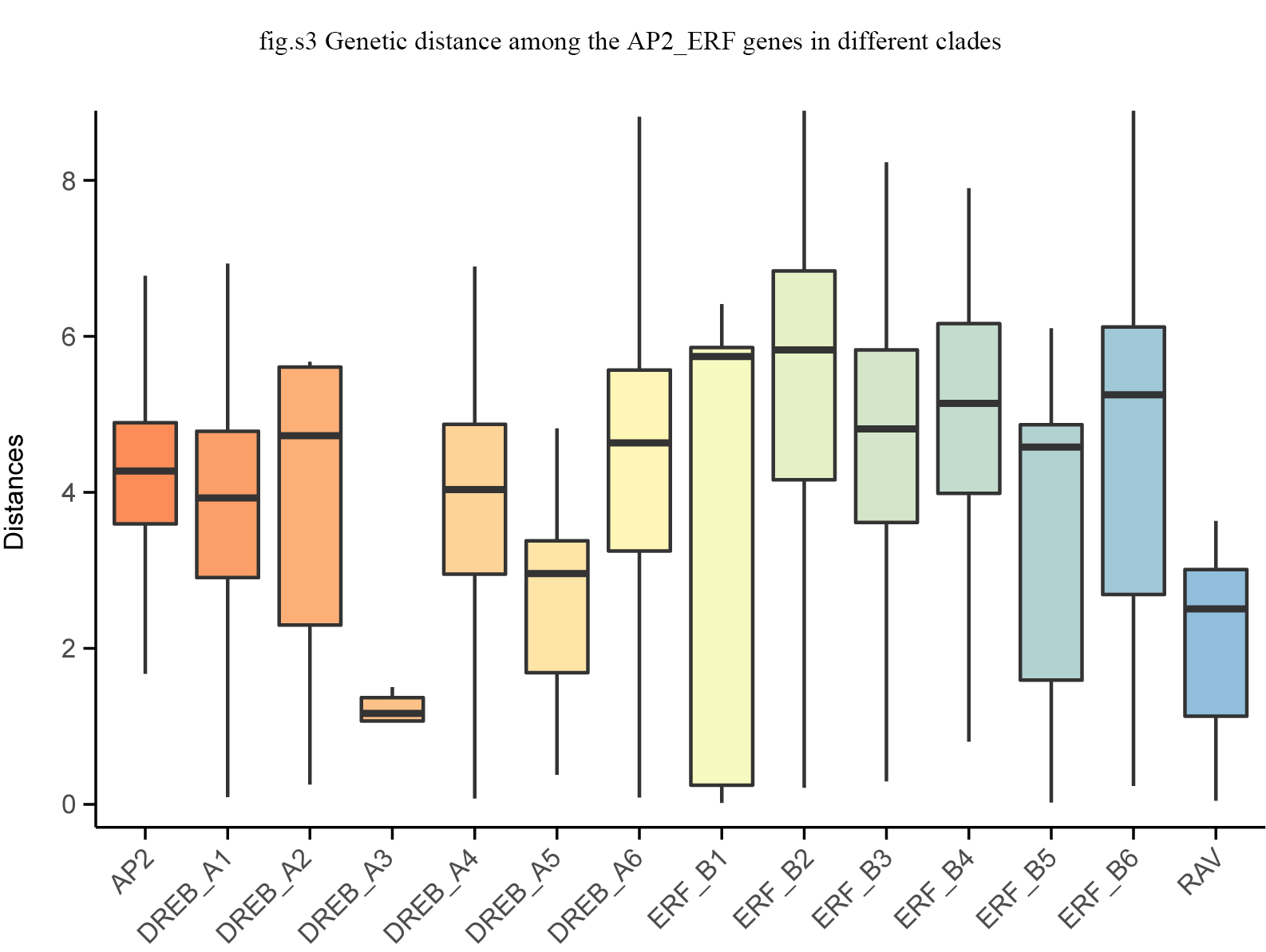

Supplement: Supplementary file 4 — Additional file 4. [file 12864_2023_9585_MOESM4_ESM.tif]
